# Length of hospital stay and readmissions after major lower extremity amputation: a Danish nationwide registry study

**DOI:** 10.2340/17453674.2024.42637

**Published:** 2024-12-23

**Authors:** Anna Trier Heiberg BRIX, Katrine Hass RUBIN, Tine NYMARK, Hagen SCHMAL, Martin LINDBERG-LARSEN

**Affiliations:** 1 Department of Orthopedic Surgery and Traumatology, Odense University Hospital, Odense, Denmark; 2 Department of Clinical Research, University of Southern Denmark, Odense, Denmark; 3 OPEN – Open Patient Data Explorative Network, Odense University Hospital and University of Southern Denmark, Odense, Denmark; 4 Department of Orthopedics and Traumatology, University Medical Center Freiburg, Freiburg, Germany

## Abstract

**Background and purpose:**

Major lower extremity amputation (MLEA) is associated with complications that may prolong length of hospital stay (LOS) and increase the risk of readmission. We primarily aimed to examine the LOS and risk of readmissions after MLEA in Denmark. Secondarily we investigated the time trends.

**Methods:**

Using Danish National Patient Registry data, this observational study analyzed 11,205 first-time MLEAs (35% transtibial amputations, 65% transfemoral amputations) performed between January 1, 2010 and December 31, 2021. Total LOS included pre- and postoperative nights. The first readmission within 30 days and 90 days post-discharge was analyzed.

**Results:**

The median total LOS after a transtibial amputation was 19 days (interquartile range [IQR] 11–30), and decreased from 28 days (IQR 17–41) in 2010 to 14 days (IQR 9–23) in 2021. The median total LOS after a transfemoral amputation was 13 days (IQR 8–22) and decreased from 16 days (IQR 9–27) in 2010 to 11 days (IQR 7–18) in 2021. Post-discharge readmission risks within 30 days were 27% (95% confidence interval [CI] 24–28) for transtibial amputations and 23% (CI 22–24) for transfemoral amputations, with corresponding 90-day risks of 40% (CI 39–42) and 35% (CI 34–36), respectively. The 30-day risk of readmission increased in both groups.

**Conclusion:**

We observed that MLEA patients’ hospital admissions lasted 2–3 weeks and decreased over the study period. A readmission risk of 23–27% within 30 days and 35–40 % within 90 days post-discharge was observed. Readmissions risk increased for both initial transtibial and transfemoral amputations over the study period.

Patients undergoing major lower extremity amputations (MLEA) are often among the most fragile in orthopedic care with an extensive comorbidity profile, a substantial risk of reoperation, and a high postoperative mortality [1,2]. Consequently, MLEA represents a high-risk intervention with significant implications for healthcare resource utilization both before and after surgery. Focusing on the escalating healthcare costs is necessary due to the ongoing expansion of the elderly population, coupled with reductions in hospital beds and staff. Prolonged hospital stays and readmissions are focal points in addressing healthcare costs [3]

Length of hospital stay (LOS) related to MLEA has been shown to be between 21 and 28 days whereas the risk of readmission has been approximated at 30% within the first month after discharge, rising to 46% in the first 6 months [4-7]. The most common causes of readmissions are stump complications and non-surgical site infections [5-7].

Both LOS and the unplanned 30-day readmissions serve as valuable metrics for assessing the healthcare burden and patient safety in various settings. Therefore, we aimed to examine LOS and risk of early readmissions after MLEA in Denmark. Furthermore, we investigated the time trends.

## Method

This study was an observational cohort study, based on data from the Danish National Health registers. The study complied with the REporting of studies Conducted using Observational Routinely-collected Data (RECORD) statement and is reported according to this [8]. Risk of reoperations and risk of mortality after MLEA in Denmark has previously been reported based on the same patient cohort [1,2]. The current study addresses LOS and readmission risk not reported earlier.

### Data sources

The Danish National Patient Registry contains data on hospitalizations and outpatient visits across Denmark, including ICD-10 diagnoses and NOMESCO surgical procedure codes, since 1977 [9]. To facilitate cross-referencing across different Danish health registries, we used the unique 10-digit social security number (CPR number). The procedure codes for amputations have not yet been validated.

The Danish National Patient Registry covers hospital contacts but excludes interactions with general practitioners, which is relevant for conditions such as diabetes that are often managed solely in primary care. For accurate diabetes classification, data from the Danish National Prescription Database were used. The Danish National Prescription Database, established in 1995, documents all reimbursed prescriptions issued in Denmark, categorized using Anatomical Therapeutic Chemical (ATC) codes, but does not include over-the-counter medications [10]. The combined use of the Danish National Patient registry and Danish National Prescription Database allows for reliable identification of diabetes cases through diagnostic codes or redeemed prescriptions for diabetic medications.

The Danish Civil Registration System holds data pertaining to individuals’ date of birth and date of death [11].

### Study population

**Figure 1:**
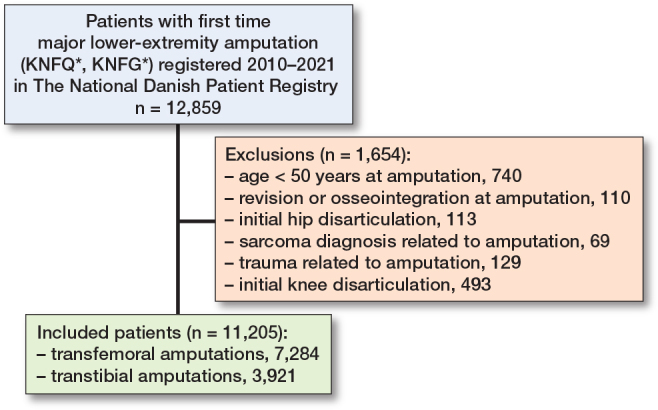
Flowchart of included patients.

The study population was defined as described in a previous paper from the research group [1]. In brief, we included patients ≥ 50 years of age, with either a primary transtibial amputation or transfemoral amputation performed between January 1, 2010 and December 31, 2021. Primary knee disarticulation (KNGQ09), primary hip disarticulation (KNFQ09), osseointegration procedures (KNFQ39*/KNFQ49* KNGQ39*/KNGQ49*), and revision procedures lacking primary amputation were excluded (Figure 1). Patients with a sarcoma diagnosis related to the amputation or a trauma diagnosis related to amputation were also excluded (see Supplementary data).

### Covariates

Age at initial MLEA was categorized into 4 groups: 50–70, 71–80, 81–90, and > 90 years.

The Charlson Comorbidity Index (CCI) was calculated using DNPR data for the 10 years preceding the procedure, following Quan et al. [12], and grouped as low (CCI score: 0), medium (CCI score: 1–2), or high (CCI score: ≥ 3). Peripheral arterial disease and diabetes were identified using data from the Danish National Patient Registry, with additional data on reimbursed diabetic medication, to ensuring inclusion of diabetes patients managed in primary care. Full definitions of comorbidities are provided (see Supplementary data).

### Outcome variables

*Length of hospital stay (LOS).* LOS was categorized into preoperative, postoperative, and total LOS, measured as the number of nights spent in the hospital, inclusive of transfers to other departments and/or hospitals.

*Readmissions.* The first readmission ≤ 30 days and ≤ 90 days was reported and included for analysis if it resulted in ≥ 1 overnight stay. Following recommendations from the Danish Health Data Authority for readmission investigations, readmissions occurring within 4 hours from discharge from the initial admission were consolidated with the initial surgical admission and included in the overall count of nights [13,14]. The time from discharge to first readmission was reported in days.

The causes of readmission were recorded by using diagnosis codes registered during the first readmission after MLEA. Patients could have multiple diagnoses registered during their first readmission, all of which were included in the analysis. Diagnoses during first readmissions were grouped into categories such as stump complications, non-surgical site infections, sepsis, and others. Surgical procedures related to MLEA during readmission were also classified as stump complications (see Supplementary data). The density of causes was explored in a density plot (Supplementary Figure 2).

### Statistics

Categorical data were reported as numbers (%), while LOS was summarized as median and interquartile range (IQR) or mean and standard deviation (SD). Readmission risks at 30 and 90 days were calculated as proportions with 95% confidence intervals (CI). A sensitivity analysis excluded patients who died during primary admission or within 30 or 90 days post-discharge (Supplementary Table 1).

Cox regression with mortality as a competing risk was used to identify factors associated with readmission at 30 and 90 days, presenting results as subdistribution hazard ratios (sHR). The applied variables were LOS ≥ 7 days, sex, initial amputation level, age group, peripheral arterial disease, diabetes, and CCI group. All variables were included in the same model. The proportional hazard assumptions were tested using Schoenfeld residuals, and found acceptable.

A multivariable logistic regression was performed to explore variables associated with a total LOS > 14 days. Variables were selected for inclusion in the multivariable model based on their clinical relevance and their theoretical association with a prolonged LOS. We included the variables sex, initial amputation level, age group, peripheral arterial disease, diabetes, and CCI group in the same model.

Time trends in LOS were analyzed with linear regression after logarithmic transformation to normalize residuals, reporting percentage changes per year.

A logistic regression model was used to test the risk of readmission over time, as this variable was binary. Assumptions for the logistic regression models were checked and found to be acceptable using goodness of fit.

All analyses were conducted in STATA v17.0 (StataCorp. 2021, Stata Statistical Software: Release 17. StataCorp LLC, College Station, TX, USA).

### Ethics, data sharing, funding, use of AI, and disclosures

Ethical approval was not required for this observational study, which was approved by the Danish Data Protection Agency (no. 21/27110). Funding was provided by the Region of Southern Denmark, Odense University Hospital and the Novo Nordisk Foundation. Data from the Danish National Patient Registry is accessible via the Danish Health Data Authority but not shareable. ChatGPT was used for minor text editing. The authors declare no conflicts of interest. Complete disclosure of interest forms according to ICMJE are available on the article page, doi: 10.2340/17453674.2024.42637

## Results

11,205 MLEAs were included, comprising in 35% (3,921) primary transtibial amputations and 65% (7,284) primary transfemoral amputations (Figure 1). The median age for transtibial amputations was 71.7 (IQR 64.2–79.4) and 77.2 (IQR 69.8–84.4) for transfemoral amputation, and the frequency of diabetes was higher among transtibial amputations (61%) compared with the transfemoral amputations (37%) (Table 1).

**Table 1:** Baseline characteristics divided into initial amputation levels. Values are count (%) unless otherwise specified

Characteristics	Transtibial amputation 3,921 (35)	Transfemoral amputation 7,284 (65)
Age, median (IQR)	71.7 (64.2–79.4)	77.2 (69.8–84.4)
Age group		
50–70	1,716 (44)	1,859 (25)
71–80	1,311 (33)	2,505 (34)
81–90	747(19)	2,259 (31)
> 90	147(3.7)	660 (9.1)
Male sex	2,754 (70)	3,994 (55)
Diabetes	2,392 (61)	2,660 (37)
Peripheral arterial disease	3,193 (83)	6,054 (81)
Both diabetes and peripheral arterial disease	1,895 (48)	2,200 (30)
Charlson Comorbidity Index		
0	766 (19)	2,220 (31)
1–2	1,905 (49)	2,874 (39)
≥ 3	1,250 (32)	2,189 (30)

### Length of hospital stay

**Figure 2:**
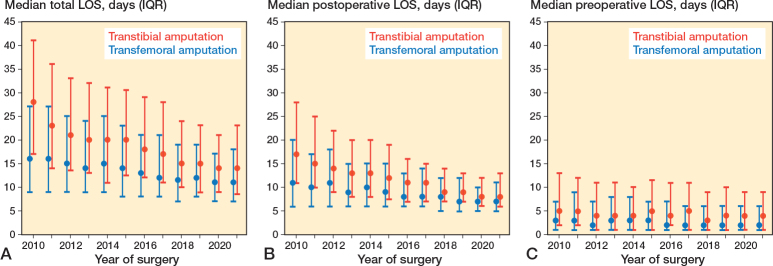
Length of hospital stay (LOS) in the study period divided into amputation levels. (A) Median total LOS with interquartile range (IQR). (B) Median postoperative LOS with IQR. (C) Median preoperative LOS with IQR.

**Figure 3:**
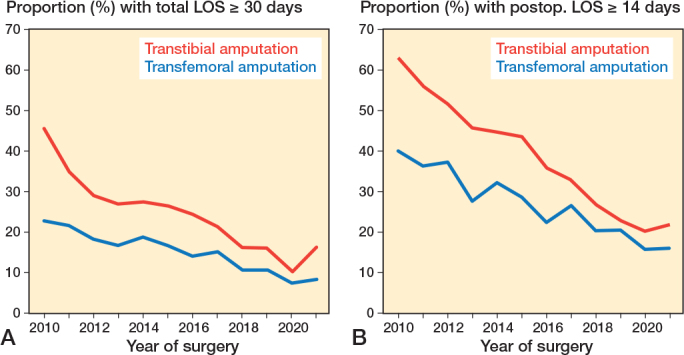
(A) Proportion of patients with a prolonged length of hospital stay (LOS) with a total LOS ≥ 30 days. (B) Proportions of patients with a prolonged postoperative LOS ≥ 14 days.

For both groups, the median total LOS decreased over time (Table 2, Figure 2). Overall, total LOS decreased significantly by 4.3% (CI –4.7 to –4.0) in a log-linear regression, and fewer patients experienced prolonged total LOS ≥ 30 days or postoperative LOS ≥ 14 days (Figure 3). 9.4% (370) in the transtibial group and 16% (1,200) in the transfemoral group died during primary admission. In the study period, the mortality during primary admission decreased from 13% to 5% for transtibial amputation and from 21% to 13% for transfemoral amputations (Supplementary Figure 1). Regression analysis showed that initial transtibial amputation was associated with a higher likelihood of total LOS > 14 days (OR 2.2, CI 2.0–2.3), while age ≥ 81 was associated with lower odds (OR 0.7, CI 0.6–0.8 for age 81–90; OR 0.4, CI 0.4–0.5 for age > 90) (Table 3). A high CCI score had no significant association with prolonged LOS.

**Table 2:** Length of hospital stay in days according to initial amputation level by amputation year. Values are median with interquartile range (upper row) and mean with standard deviation (lower row)

LOS	2010	2011	2012	2013	2014	2015	2016	2017	2018	2019	2020	2021	Total
Transtibial amputation													
Total	28 (17–42)	23 (14–36)	21 (13–33)	20 (13–32)	20 (11–31)	20 (12–31)	18 (12–29)	17 (11–28)	15 (10–24)	15 (9–24)	14 (9–21)	14 (8–23)	19 (11–30)
	33 (24.6)	29.5 (24.2)	26.5 (21.0)	25.8 (23.8)	24.7 (22.1)	23.8 (16.9)	23.1 (19.5)	22.2 (17.5)	19.9 (18.2)	19.9 (21.9)	17.8 (17.1)	18.4 (15.1)	24.2 (21.1)
Preop.	5 (2–13)	5 (2–12)	4 (1–11)	4 (1–11)	4 (1–10)	5 (1–12)	4 (2–11)	5 (1–11)	3 (1–9)	4 (1–11)	4 (1–9)	4 (1–9)	4 (1–11)
	9.8 (12.8)	9.1 (12.0)	8.5 (11.3)	8.1 (10.7)	7.7 (11.0)	8.2 (10.3)	7.9 (9.3)	8.1 (11.2)	7.4 (12.2)	8.1 (13.9)	6.4 (8.1)	7.4 (9.6)	8.1 (11.2)
Postop.	17 (11–29)	15 (10–25)	14 (9–22)	13 (8–20)	13 (8–20)	12 (7–19)	11 (7–16)	11 (7–15)	9 (7–14)	9 (7–13)	8 (6–12)	8 (6–13)	11 (7–19)
	23.2 (21.4)	20.4 (19.7)	18 (15.2)	17.7 (20.3)	17 (16.6)	15.6 (12.8)	15.2 (15.2)	14.1 (12.1)	12.4 (9.9)	11.8 (11.3)	11.5 (13.7)	11 (11.4)	16.1 (16.1)
Transfemoral amputation													
Total	16 (9–27)	16 (9–27)	15 (9–25)	14 (9–24)	15 (9–25)	14 (8– 23)	13 (8–21)	12 (8–21)	11.5 (7–19)	12 (8–19)	11 (7–17)	11 (7–18)	13 (8–22)
	21.5 (18.4)	21.9 (20.1)	20.7 (19.6)	19.7 (18.2)	20.7 (19.3)	19 (19.2)	17.6 (16.6)	17.6 (15.1)	16.3 (21.5)	16.2 (16.4)	14.4 (12.8)	15.1 (20)	18.1 (18.3)
Preop.	3 (1–7)	3 (1–9)	2 (1–7)	3 (1–8)	3 (1–8)	3 (1–7)	2 (1–7)	2 (1–6)	2 (1–6)	2 (1–6)	2 (1–6)	2 (1–6)	3 (1–7)
	6.5 (10.0)	7.1 (10.2)	6.0 (10.3)	6.3 (9.0)	7.1 (11.1)	5.6 (8.9)	6.0 (10.6)	5.2 (7.4)	5.0 (6.9)	5.1 (7.5)	5.4 (9.1)	5.1 (8.1)	5.8 (9.1)
Posto.	11 (6–20)	10 (6–17)	11 (6–18)	9 (6–15)	10 (6–15)	9 (6–15)	8 (6–13)	8 (6–14)	8 (5–12)	7 (5–12)	7 (5–10)	7 (5–11)	8 (6–14)
	14.9 (14.6)	14.9 (15.7)	14.7 (14.8)	13.4 (15.3)	13.6 (14.3)	13.4 (15.8)	11.6 (11.5)	12.4 (12.6)	11.4 (19.5)	11.1 (13.5)	9 (8.2)	10 (15.5)	12.3 (14.6)

**Table 3:** Potential factors associated with a total length of hospital stay > 14 days analyzed in a multivariable logistic regression model

Factor	Odds ratio (CI)
Index amputation level	
Transfemoral	1 (ref.)
Transtibial	2.2 (2.0–2.3)
Sex	
Male	1 (ref.)
Female	1.0 (0.9–1.1)
Peripheral arterial disease	
No	1 (ref.)
Yes	1.1 (0.9–1.3)
Diabetes	
No	1 (ref.)
Yes	1.2 (1.1–1.3)
Age group	
50–70	1 (ref.)
71–80	1.0 (0.9–1.1)
81–90	0.7 (0.6–0.8)
> 90	0.4 (0.4–0.5)
CCI group	
0	1 (ref.)
1–2	1.0 (0.9–1.1)
≥ 3	1.1 (0.9–1.2)

#### LOS after transtibial amputation

The overall median total LOS for transtibial amputations was 19 days (IQR 11–30), while the overall mean LOS was 24.2 days (SD 21.1) (Table 2). The median total LOS for transtibial amputations decreased from 28 days (IQR 17–41) in 2010 to 14 days (IQR 8.5–23) in 2021. In the study period, the decrease in total LOS after transtibial amputation was explored by a log-linear regression model and was significant at 5.0%/year (CI –4.4 to –5.6). The overall median preoperative LOS was 4 days (IQR 1–11) while the median postoperative LOS was 11 days (IQR 7–19).

#### LOS after transfemoral amputation

The overall median total LOS for transfemoral amputations was 13 days (IQR 8–22), while the overall mean LOS was 18.1 days (SD 18.3) (Table 2). The median total LOS for transfemoral amputations decreased from 16 days (IQR 9–27) in 2010 to 11 days (IQR 7–18) in 2021. In the study period, the decrease in total LOS after transfemoral amputation was 3.3%/year (CI –2.8 to –3.7). The overall median preoperative LOS was 3 days (IQR 1–7) while the median postoperative LOS was 8 days (IQR 6–14).

### Readmissions

**Figure 4:**
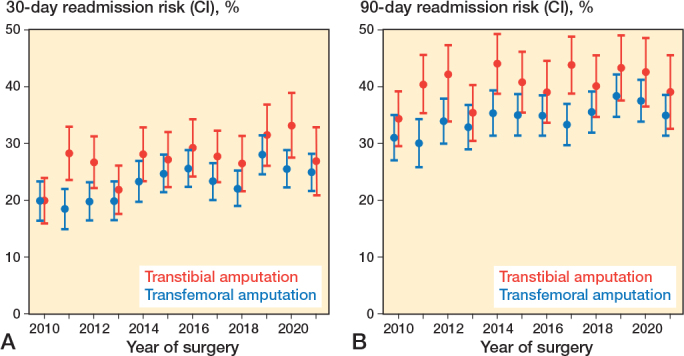
Risk of readmission from 2010 to 2021 with 95% confidence interval (CI).

In the study period the risk of readmission within 30 days was rising for both groups (Figure 4). Predictors for readmission were investigated with Cox regression analysis with mortality as a competing risk (Table 4). Significant variables with higher sHR for readmission at 30 days were: total LOS > 7 days sHR 1.5 (CI 1.4–1.7), transtibial amputation sHR 1.1 (1.0–1.2), female sex sHR 1.1 (1.0–1.2), peripheral arterial disease sHR 1.2 (1.1–1.4), and a high CCI score sHR 1.3 (1.2–1.5). For 90-day readmission, the results were similar.

**Table 4:** Potential risk factors associated with readmission at 30 days and 90 days analyzed in a multivariable Cox regression model with mortality as competing risk

Factor	30-day readmission sHR (CI)	90-day readmission sHR (CI)
Total length of stay		
≤ 7 days	1 (ref.)	1 (ref.)
> 7 days	1.5 (1.4–1.7)	1.4 (1.3–1.6)
Index amputation level		
Transfemoral	1 (ref.)	1 (ref.)
Transtibial	1.1 (1.0–1.2)	1.1 (1.1–1.2)
Sex		
Male	1 (ref.)	1 (ref.)
Female	1.1 (1.0–1.2)	1.1 (1–1.2)
Peripheral arterial disease		
No	1 (ref.)	1 (ref.)
Yes	1.2 (1.1–1.4)	1.3 (1.2–1.4)
Diabetes		
No	1 (ref.)	1 (ref.)
Yes	1.0 (0.9–1.1)	1.0 (0.9–1.1)
Age group		
50–70	1 (ref.)	1 (ref.)
71–80	1.0 (0.9–1.1)	1.0 (0.9–1.1)
81–90	1.0 (0.9–1.1)	1.0 (0.9–1.0)
> 90	0.9 (0.7–1.0)	0.8 (0.7–0.9)
CCI group		
0	1 (ref).	1 (ref.)
1–2	1.1 (1.0–1.3)	1.1 (1.0–1.2)
≥ 3	1.3 (1.2–1.5)	1.3 (1.2–1.4)

#### Readmission after transtibial amputation

**Figure 5:**
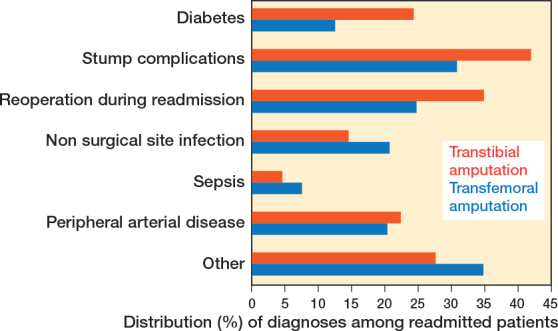
Readmission causes divided into categories. All registered diagnoses during first readmission were included. Figure represents percentage of readmitted patients.

For transtibial amputations, 27% (CI 25–28) were readmitted within 30 days and 40% (CI 40–42) within 90 days (Table 5). The 30-day readmission risk increased annually by 3.4% (CI 1.2–5.4), while the 90-day risk showed no significant trend (1.7%/year, CI –1.7 to 3.6). Median time to readmission was 19 days (IQR 8–41). Of the readmissions, 42% were attributed to stump complications, though not all required surgery during the first readmission. Diabetes (24%) and peripheral arterial disease (22%) were also common causes of readmission, followed by non-surgical site infections (15%) and sepsis (4.5%) (Figure 5). Stump complication-related readmissions frequently occurred within 30 days post-discharge (density plot, see Supplementary Figure 2).

**Table 5:** Risk of readmission within 30 and 90 days after discharge from primary admission according to initial amputation level by amputation year

Year	Transtibial amputation			Transfemoral amputation		
	n/N	30-day readmission risk % (CI)	90-day readmission risk % (CI)	n/N	30-day readmission risk % (CI)	90-day readmission risk % (CI)
2010	76/381	20 (16–24)	34 (30–39)	101/509	20 (16–23)	31 (27–35)
2011	99/351	28 (24–33)	41 (35–46)	83/449	19 (15–22)	30 (26–34)
2012	96/360	27 (22–31)	42 (37–47)	107/542	20 (16–23)	34 (30–38)
2013	80/367	22 (18–26)	35 (31–40)	108/544	20 (17–23)	33 (29–37)
2014	98/349	28 (23–33)	44 (39–49)	127/546	23 (20–27)	35 (31–39)
2015	89/328	27 (22–32)	41 (36–46)	161/654	25 (21–28)	35 (31–38)
2016	91/312	29 (24–34)	39 (34–45)	178/696	27 (22–29)	35 (31–39)
2017	106/383	28 (23–32)	44 (39–49)	153/657	23 (20–27)	33 (30–37)
2018	83/314	26 (22–31)	40 (35–46)	148/672	22 (19–25)	36 (32–39)
2019	92/293	31 (26–38)	43 (38–49)	185/661	28 (25–31)	38 (35–42)
2020	87/263	33 (27–39)	43 (37–49)	171/671	26 (22–29)	38 (34–41)
2021	59/202	27 (21–33)	39 (33–46)	170/683	25 (22–28)	35 (31–39)
Total	1,056/3,921	27 (26–28)	40 (39–42)	1,692/7,284	23 (22–24)	35 (34–36)

n/N = number readmitted at 30 days/total number

#### Readmission after transfemoral amputation

For transfemoral amputations, 23% (CI 22–24) were readmitted within 30 days, rising to 35% (CI 34–36) within 90 days (Table 5). Readmission risks increased annually by 3.5% (CI 1.9–5.2) at 30 days and 2.2% (CI 0.8–3.6) at 90 days. Median time to readmission was 18 days (IQR 7.5 to 40). Stump complications accounted for 31% of readmissions, followed by non-surgical site infections (21%), peripheral arterial disease (20%), diabetes (12%), and sepsis (7.7%) (Figure 5). Non-surgical site infections were predominantly within 30 days post-discharge (density plot, see Supplementary Figure 2).

## Discussion

We aimed to examine the length of hospiyal stay (LOS) and risk of readmissions after MLEA in Denmark. We found a decrease in the length of stay (LOS) for MLEA without concurrent changes in the risk of readmissions within ≤ 90 days. However, the total LOS remained notably high and an increase in readmission risk was observed, exerting a substantial impact on hospital resources. Stump complications were the most frequent complication associated with readmissions.

### Length of hospital stay (LOS)

LOS was unexpectedly longer after transtibial amputations compared with transfemoral amputations, despite less surgical stress and younger patients. Possible explanations include higher risks of stump complications and re-amputations at more distal levels [2], or that transfemoral patients often reside in nursing homes or have better in-home support, facilitating earlier discharge [6,15]. Prosthesis candidates may also experience longer LOS due to initiation of rehabilitation during admission. A nationwide study from England reported postoperative median LOS of 21–28 days after MLEA, and in contrast to our findings they found longer hospitalizations after above-knee amputations, and in patients with diabetes [16]. Another English nationwide study indicated an overall total median LOS at 33 days, decreasing with no preoperative attempts at limb-saving interventions [17].

These differences may be explained by differences in study periods or more likely by healthcare and rehabilitation variations between Denmark and England. The decrease in LOS during our study was mainly due to fewer patients with prolonged stays (≥ 14 days). Stable preoperative LOS suggests persistent delays in amputation timing, possibly due to challenges in diagnosing critical limb ischemia or scheduling surgeries, potentially worsening outcomes [17]. Hence, improving the preoperative phase could enable more patients to be treated electively without preoperative hospitalization.

Patients aged 81 and above had a significantly lower risk of LOS ≥ 7 days, while higher comorbidity was not associated with longer stays. This was unexpected, as older patients are usually more frail and would be expected to have a higher LOS. This suggests older patients might be discharged more efficiently due to established in-home support or nursing home residency.

Despite reductions, LOS after MLEA remains high compared with other orthopedic procedures like hip fractures (median LOS of 8 days in 2014) [18] or hip and knee arthroplasties (median LOS of 1–2 days between 2010 and 2020) [19]. Matching LOS with elective arthroplasty procedures is unrealistic due to patient differences, but aiming for LOS similar to hip fractures may be feasible. However, Kayssi et al. [7] identified a LOS ≤ 7 days as a risk factor for 30-day readmission after MLEA, suggesting a potential threshold where the benefits of a shorter LOS may be outweighed by adverse outcomes. Our results suggest the opposite, with a LOS > 7 days associated with a higher risk of readmission at both 30 and 90 days in a competing risk analysis, probably because patients leaving the hospital earlier tend to be healthier and more self-sufficient. Prolonged hospitalizations may also increase the risk of hospital-acquired conditions, resulting in no added benefit to the patients. However, the optimal LOS threshold remains under debate.

### Risk of readmission

The 30-day readmission risk of 23–27% found in our study was similar to the readmission risk reported in studies conducted in the USA and Canada by Phair et al. and Kayssi et al. [5,7]. Another study from USA by Curran et al. found a lower 30-day readmission risk at 18% but only included patients from 2011–2012 and minor amputations, which might explain the difference [6]. The readmission risk from our study aligns with the results in the above-mentioned studies from the USA and Canada, which consolidate the theory that MLEA patients are at high risk for readmission.

The most common causes of readmissions included stump complications and non-surgical site infections [5-7]. Stump complications were the most common reason for readmission in our study, accounting for 42% of readmitted transtibial amputation patients and 31% of readmitted transfemoral amputation patients. This aligns with literature findings where stump complications represent the primary readmission cause after MLEA, with rates of stump complications related to readmissions ranging from 14–49% [5-7]. Variations in classification, registration, and coding might explain the relatively broad range.

Patients with initial transfemoral amputation had a higher frequency of non-surgical site infections and non-wound related readmission causes, which also aligns with other studies [5,6].

We found that the risk of readmission after MLEA significantly increased in the first 30 days post-discharge. For improvement of the readmission risk after MLEA, a focus in particular on stump complications, non-surgical site infections, and complications of diabetes and peripheral arterial disease seems to be a good approach.

### Strengths and limitations

This study used nationwide data from the Danish health registers, which are known for high quality and comprehensive coverage, overall minimizing information bias. However, the procedure codes for amputations have not yet been validated.

Limitations include the lack of lifestyle data (e.g., frailty, physical activity, smoking, BMI), which introduces potential confounding. Additionally, the registry does not contain the discharge destinations or prior in-home assistance, factors that may influence LOS. Although we excluded patients with sarcoma and trauma diagnoses, the exact cause of and indication for MLEA remains unclear, adding potential bias. Due to the observational design and residual confounding, associations between variables, LOS, and readmissions should be interpreted with caution.

### Conclusion

We observed that MLEA patients had hospital admissions lasting 2–3 weeks in combination with readmission risk of 23–27% within 30 days and 35–40% within 90-days post discharge. Although LOS significantly decreased over the study period, the risk of readmissions in the first 30 days was increasing. The most common cause of readmission was stump complications for both initial transtibial and transfemoral amputations. Other relevant readmission causes were non-surgical site infections, diabetic complications, and complications related to peripheral arterial disease, and prevention of these could potentially lower the readmission risk. Our findings highlight the significant impact of MLEA patients on hospital resource consumption and the need for improvements in perioperative patient care.

*In perspective,* the integration of orthogeriatric care models, with inspiration from those established for hip fracture management [20-22], into the pre-, peri- and postoperative care of MLEA patients, combined with more specialized surgeons, could be a future direction in the treatment of this fragile patient group. Additionally, initiatives to streamline care protocols, enhance multidisciplinary coordination, and improve patient selection for surgery could further improve the treatment of such patients.

### Supplementary data

Supplemental Tables, Figures, and code definitions are available as supplementary data on the article page, doi: 10.2340/17453674.2024.42637

## Supplementary Material



## Notes

ATHB and MLL designed the study protocol. ATHB conducted the data management and analysis. ATHB drafted the manuscript, first revised critically by MLL, then KHR, TN, and HS thereafter. All authors approved the final manuscript.

The authors acknowledge OPENRegistry & Statistics for their insight in data management and statistics advice.

Handling co-editors: Bart A Swierstra and Philippe Wagner

*Acta* thanks Pelle Gustafson and other anonymous reviewers for help with peer review of this manuscript.
