# Supplementary material for: Length of hospital stay and readmissions after major lower extremity amputation: a Danish nationwide registry study
Source: Acta Orthop. 2024 Dec 23;95:737–43. doi: 10.2340/17453674.2024.42637 (PMC11664435; doi:10.2340/17453674.2024.42637)
Supplement: Supplementary file 1 [file ActaO-95-42637-s1.pdf]

Supplementary information

This appendix addresses the definition of variables.

Amputation codes

Primary major amputations

Above knee amputation

Hip disarticulation	KNFQ09
Transfemoral amputation	KNFQ19
Other amputation on the femur/hip	KNFQ99

Below knee amputation

Knee disarticulation	KNGQ09
Transtibial amputation	KNGQ19
Other amputation on knee/tibia	KNGQ99

Revision codes

KNGQ29	Stump revision knee/lower leg
KNGQ29A	Stump revision after knee disarticulation
KNGQ29B	Stump revision after transtibial amputation
KNFQ29	Stump revision hip/tight
KNFQ29A	Stump revision after hip disarticulation
KNFQ29B	Stump revision after transfemoral amputation

If 1 of the above listed revision codes or 1 of the unspecific procedure codes for wound revisions: **KQDB*, KTNG05 or KNFW*** were present in the first readmission it was interpreted as the patient had a reoperation during the readmission.

Osseointegration codes were excluded**:** KNGQ39, KNGQ49 KNFQ39, KNFQ49

A case was excluded in case of a revision code without a prior primary amputation code.

KNGQ99/KNFQ99 were grouped as Transtibial amputation (KNGQ19) or Transfemoral amputation (KNFQ19,) respectively.

Sarcoma exclusion criteria: A case was excluded if a sarcoma diagnosis (DC40*, DC41* or DC49*) was registered ±1 year from index surgery.

Trauma exclution criteria: An amputation was classified as trauma associated, and then excluded if one of the following ICD10 codes were present in relation to the amputation: DS980 (traumatic amputation, foot), DS88* (traumatic amputation, knee), DS78* (traumatic amputation, hip), DT136 (traumatic amputation, lower extremity, unspecified), DT05, DT053-6, DT058-9 (traumatic amputation, in combination), DT036 (sequelae after traumatic amputation, lower extremity).

Definition for diagnoses, ICD10

Diabetes

A patient was categorized with diabetes if one of the following ICD10 codes were registered: E10*, E11*, E13*, E14* or the patient has redeemed 2 or more anti-diabetic medicine prescriptions in the same ATC group with the following ATC codes 5 years before the index date: A10A (Insulins and analogs) A10B (Blood Glucose lowering drugs, excl. insulins), A10X (Other drugs used in diabetes).

Arteriosclerosis/PAD

Arteriosclerosis/Peripheral arterial disease (PAD) was defined as one or more of the following ICD10 codes: I70, I702, I709, I739A, I739C or I743

Readmissions

A patient could have more than one diagnosis registered during readmission

Definition of readmission causes

Stump complications	Either one of the following:<br>DT87*, DT814, DT819, DT813, DT810, DL024, DS81*, DS913, DS711, SM769, DG546, DG547, DR529, DR029, DZ47*, DZ48*, DA480, DL089, DL088, DL979, DT888, DT889, DT930, DT939, DT131<br>*or*<br>Re-operation during first readmission with one of the following procedure codes:<br>KNGQ*, KNFQ*, KQBD*, KTNG05, KNFW*
Non-surgical site infection	Infection: DA49*, DR509<br>Pneumonia: DJ15*, DJ17*, DJ18*, DJ229, DJ168<br>Urinary tract infection: DN390, DN300, DN308C, DN309, DN308
Sepsis	DA419, DA419B, DA419C, DR572, DA40*. DA41*
Diabetes	DE10*, DE11*, DE13*, DE14*, DE162
Peripheral arterial disease	DI70*, DI738, DI739
Other	None of the above

Supplemental figures and tables

Supplemental Figure 1. Mortality during primary admission

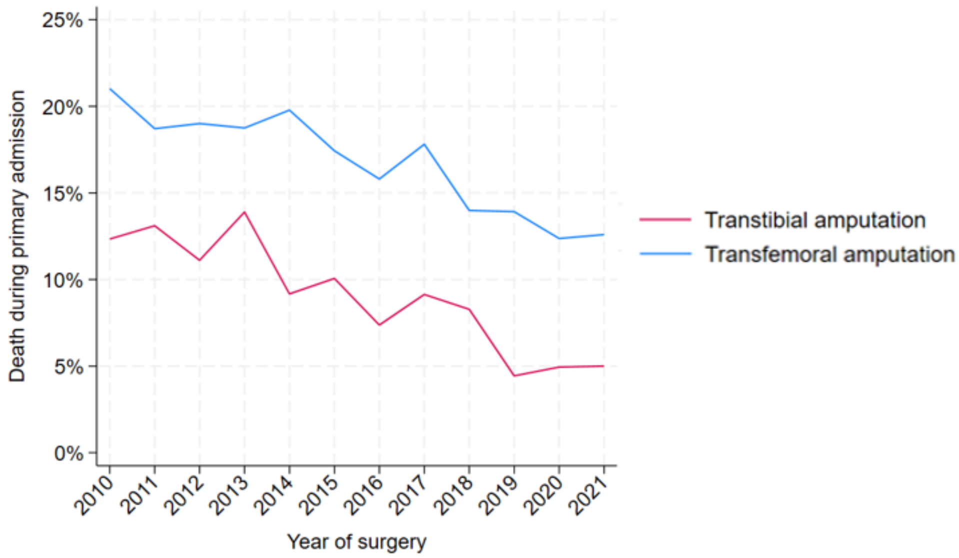

Supplemental Figure 2. Density plots

A) Transtibial Amputation

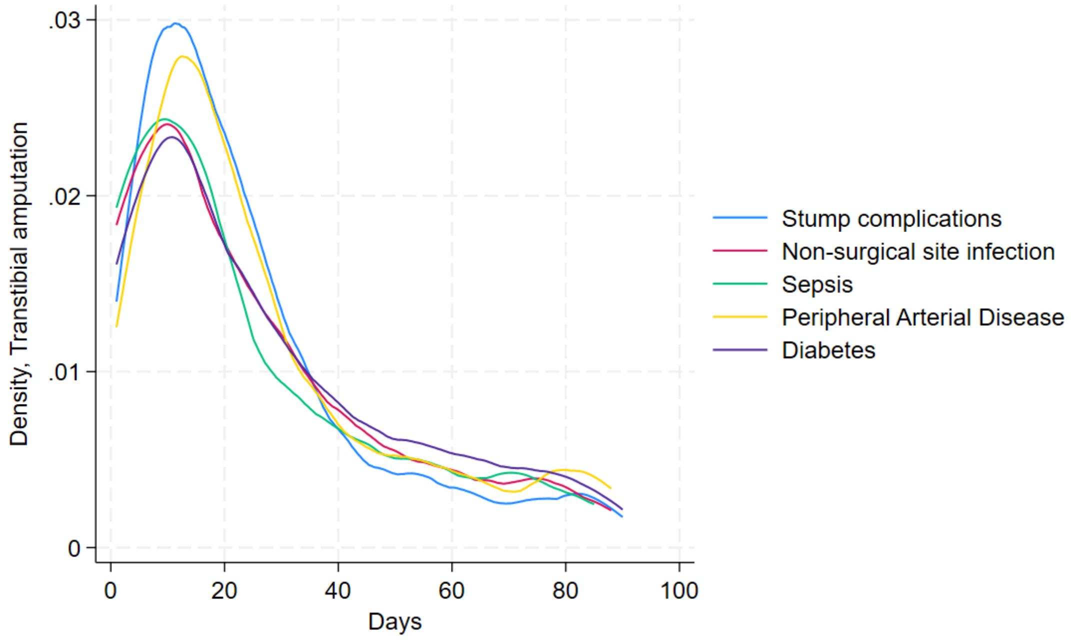

B) Transfemoral amputation

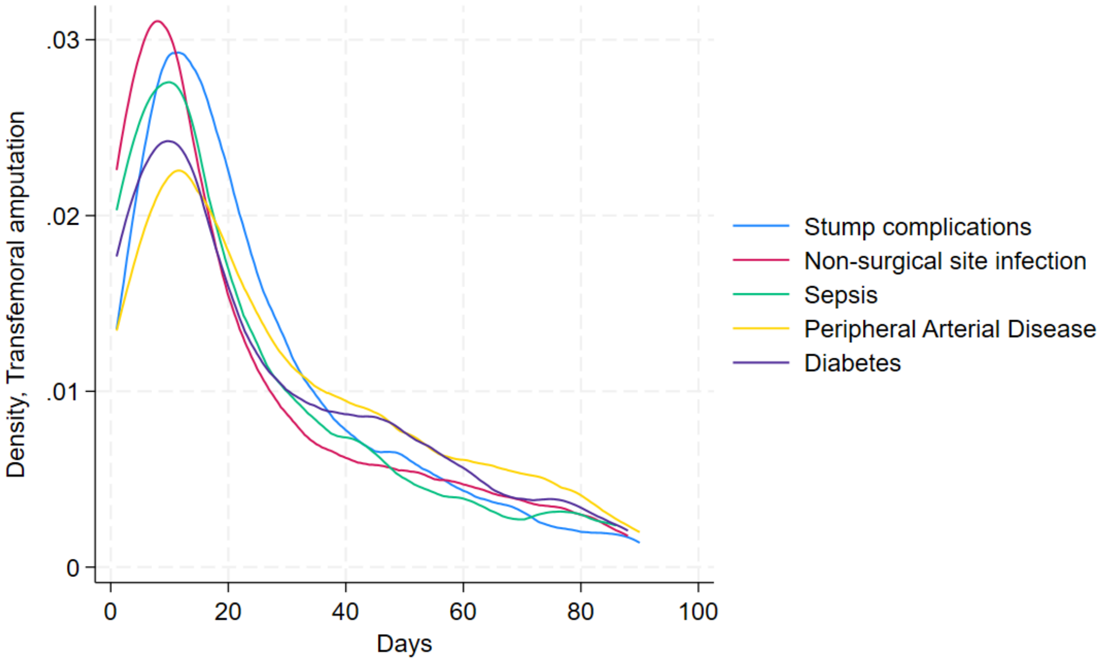
Density, Transfemoral amputation
.03
.02
.01
0
0
20
40
60
80
100
Days
Stump complications
Non-surgical site infection
Sepsis
Peripheral Arterial Disease
Diabetes

Supplemental Table 1. Risk of readmission

**When death during primary admission is excluded**		
	Transtibial amputation<br>n = 6,084	Transfemoral amputation<br>n = 3,551
30-day readmission risk	27.8% (26.6–28.10)	29.7% (28.2–31.4)
90-day readmission risk	41.5% (40.2–42.7)	44.6% (42.0–46.3)
**When only those who were at risk the whole period were included in analysis**		
30-day readmission risk for persons alive 30 days after discharge	Alive 30 days after discharge: 3,380<br>Readmitted: 976 (28.9%)	Alive 30 days after discharge: 5,339<br>Readmitted: 1,413 (26.5%)
90-day readmission risk for persons alive 90 days after discharge	Alive 90 days after discharge: 3,203<br>Readmitted: 1,353 (42.2%)	Alive 90 days after discharge: 4,716<br>Readmitted: 1,769 (37.5%)

Supplemental Table 2. Overview of diagnosis codes registered during the first readmission. The percentage refer to percent of readmitted MLEA patients, not the entire included cohort

	Transtibial amputation	Transfemoral amputation
Number of readmitted patients	1,584	2,524
Stump complications	665 (42)	779 (31)
Reoperation	553 (35)	625 (25)
Cardiovascular	185 (12)	314 (12)
Dehydration/electrolyte derangement	78 (4.9)	174 (6.9)
Anemia	47 (3.0)	84 (3.3)
Gastroenterology	174 (11)	284 (11)
Rheumatic	37 (2.3)	71 (2.8)
Pulmonary	81 (5.1)	213 (8.4)
Kidney	144 (9.1)	154 (6.1)
Cerebral	38 (2.4)	113 (4.5)
Diabetes	385 (24)	317 (13)
Pulmonary embolism	14 (0.9)	38 (1.5)
Sepsis	72 (4.5)	191 (7.6)
Non-surgical site infection	231 (15)	522 (21)
Peripheral arterial disease	355 (22)	514 (20)
Unspecified	204 (13)	445 (18)
